# Kit ligand promotes first polar body extrusion of mouse preovulatory oocytes

**DOI:** 10.1186/1477-7827-7-26

**Published:** 2009-04-03

**Authors:** Yinghui Ye, Kazuhiro Kawamura, Mitsue Sasaki, Nanami Kawamura, Peter Groenen, Maarten D Sollewijn Gelpke, Rami Rauch, Aaron JW Hsueh, Toshinobu Tanaka

**Affiliations:** 1 Department of Obstetrics and Gynecology, Akita University School of Medicine, Akita, Japan; 2 Department of Reproductive Endocrinology, Women's Hospital, Zhejiang University School of Medicine, Zhejiang, PR China; 3 Department of Dermatology and Plastic Surgery, Akita University School of Medicine, Akita, Japan; 4 Organon, Oss, the Netherlands; 5 Division of Reproductive Biology, Department of Obstetrics and Gynecology, Stanford University School of Medicine, Stanford, California, USA

## Abstract

**Background:**

Shortly after stimulation by the preovulatory surge of luteinizing hormone (LH), oocytes arrested at the late prophase I resume meiosis characterized by germinal vesicle breakdown (GVBD), chromosome condensation, and extrusion of the first polar body in preparation for fertilization and early embryonic development. However, oocytes express few or no LH receptors and are insensitive to direct LH stimulation. Thus, factors released by granulosa or theca cells expect to convey the LH stimuli to oocytes. To identify candidate ligand-receptor pairs potentially involved in the process of oocyte maturation, we performed DNA microarray analyses of ovarian transcripts in mice and identified Kit ligand (Kitl) as an ovarian factor stimulated by the LH/hCG surge. The purpose of this study is to investigate the roles of KITL in the nuclear and cytoplasmic maturation of preovulatory mouse oocytes.

**Methods:**

The levels of Kitl and c-kit transcripts in mouse ovaries and isolated ovarian cells were determined by real-time RT-PCR, while expression of KITL protein was examined by immunohistochemistry. Follicle culture, cumulus-oocyte complexes (COC) and denuded oocytes culture were used to evaluate the effect of KITL on mouse oocyte nuclear maturation. To assess the effect of KITL treatment on the cytoplasmic maturation of preovulatory oocytes, we performed in vitro maturation of oocytes followed by in vitro fertilization.

**Results:**

Major increase of Kitl transcripts in granulosa cells and mouse ovaries, and predominant expression of c-kit in preovulatory oocytes were identified by real-time RT-PCR. Predominant expression of KITL protein was found in granulosa cells of preovulatory and small antral follicles at 4 h after hCG treatment. In vitro cultures demonstrated that treatment with KITL enhanced first polar body extrusion in a dose-dependent manner. Moreover, treatment of COC with KITL enhanced first polar body extrusion with increase in cyclin B1 synthesis which is important for the progression of meiotic maturation after GVBD. In contrast, treatment of cultured preovulatory follicles with KITL did not affect GVBD and KITL has no effect on cytoplasmic maturation of preovulatory oocytes.

**Conclusion:**

Our findings suggest potential paracrine roles of KITL in the nuclear maturation of preovulatory oocytes by promoting first polar body extrusion.

## Background

Meiosis of mammalian oocytes is characterized by prolonged arrest at the diplotene stage of prophase I. Before ovulation, preovulatory oocytes undergo maturation. Oocyte maturation includes nuclear maturation events characterized by germinal vesicle breakdown (GVBD) and the extrusion of first polar body [1]. In addition to nuclear maturation to complete the first meiotic division, oocytes also undergo cytoplasmic maturation characterized by cytoplasmic changes essential for monospermic fertilization, processing of the sperm, and preparation for development to preimplantation embryos [2,3]. The preovulatory luteinizing hormone (LH) surge triggers oocytes to resume meiosis characterized by GVBD, chromosome condensation, and extrusion of the first polar body in preparation for fertilization and early embryonic development. However, oocytes express few or no LH receptors and are insensitive to direct LH stimulation. Thus, factors released by the LH-responsive granulosa or theca cells likely convey the LH stimuli to oocytes [4]. Recent studies demonstrated that the endocrine hormone LH stimulates ovarian production of epidermal growth factor (EGF)-like factors from granulosa cells and insulin-like 3 (INSL3) from theca cells to promote GVBD [5,6]. Preovulatory increases in LH also stimulate granulosa cells to produce brain-derived neurotrophic factor (BDNF) that is capable of promoting first polar body extrusion and the cytoplasmic maturation of oocytes [7].

We performed DNA microarray analyses using ovaries from immature mice following gonadotropin stimulation and identified candidate ligand-receptor pairs potentially involved in the process of oocyte maturation [7]. Among different candidate genes, we found major increases in the ovarian expression of kit ligand (Kitl, also known as stem cell factor) during the preovulatory period. KITL is a pluripotent growth factor which is important in the differentiation and growth of diverse stem cell lineages including hematopoietic stem cells, neuroblasts, primordial germ cells, et al [8] and its receptor is c-kit (CD117), a receptor protein tyrosine kinase belonging to the platelet-derived growth factor receptor family [9]. KITL and c-kit are the products of Steel (Sl) and White Spotting (W) loci in mice, respectively. Mutations in these loci will lead to infertility due to impairment of primordial germ cell survival, migration and proliferation, as well as defective follicle development [8]. KITL exists as both soluble (KITL1) which mediates cellular migration and membrane-bound (KITL2) proteins which contributes to cell survival and proliferation[8]. Both KITL1 and KITL2 transcripts can be detected in ovaries and recent study showed that KITL2, but not KITL1 promoted mouse oocyte growth[10]. Although the KITL/c-kit signaling pathway is essential for diverse ovarian processes, including the initiation of primordial follicle development [11,12], growth of oocytes in primordial and pre-antral follicles [11,13-16], proliferation of granulosa cells [17], recruitment of theca cells, and the regulation of ovarian steroidogenesis [8,14,18,19], few studies have explored the roles of KITL in oocyte maturation.

Here, we demonstrated preovulatory increases of KitL transcripts in ovarian granulosa and cumulus cells, but not its receptor, c-kit, which is predominantly expressed in mouse oocytes. Treatment of cultured oocytes with KITL enhanced first polar body exclusion.

## Method

### Animals

Ovarian samples were obtained from immature female B6D2F1 mice at 25–27 days of age (CLEA Japan, Inc., Tokyo, Japan) after treatment with a single intraperitoneal injection of 7 IU pregnant mare serum gonadotropin (PMSG; Calbiochem, Cambridge, Massachusetts, USA), followed at 48 h later with 10 IU of human chorionic gonadotropin (hCG, ASKA Pharmaceutical, Co., Ltd, Tokyo, Japan) to stimulate follicle maturation and ovulation, respectively. The care of animals was approved by the Animal Research Committee, Akita University School of Medicine.

### DNA microarray analysis

Mice (n = 108) were injected at 21 days of age with Humegon (7.5 IU per animal, Organon, Oss, Netherlands) containing FSH and LH activities to stimulate follicular growth. Forty-eight hours later, some animals were treated ip with Pregnyl (5 IU per animal, Organon) containing LH activity to induce ovulation. Ovaries were dissected from animals killed bi-hourly after Humegon treatment (three mice per group) and hourly after Pregnyl treatment (one mouse per group) for RNA extraction (TRIzol, Invitrogen Corp., Carlsbad, CA). Aliquots of 6 μg of total RNA at 1 μg/μl for one-chipset hybridization were stored at -80 C. Samples were hybridized to the Affymetrix mouse MGU74v2 arrays A, B, and C according to standard Affymetrix protocols. The pooled follicular phase samples were hybridized in duplicate, and the post Pregnyl samples were single determinations [7].

### Quantitative real-time RT-PCR

For quantitative real-time RT-PCR, oocytes, cumulus cells, and mural granulosa cells were collected from ovaries of PMSG-primed immature mice at 48 h after treatment or from ovaries of PMSG-primed immature mice at 2 h after hCG treatment. Cumulus-oocyte complexes (COCs) were obtained by puncturing the largest follicles of preovulatory ovaries, and denuded oocytes (DOs) were separated from cumulus cells by mechanical pipetting. Granulosa cells were obtained separately by puncturing preovulatory follicles, followed by the removal of COCs.

Quantitative real-time RT-PCR of transcript levels were performed using whole ovaries and isolated ovarian cells using a Smartcycler (Takara, Tokyo, Japan) [6,7]. The primers and hybridization probes for real-time PCR of Kitl, c-kit and histone H2a are as follow: Kitl: sense 5'-ACATGGTGGCTGCTCTTCTT-3', antisense 5'-TGCAGACCTGATAGCATTGG-3', probe 5'-6-carboxy-fluorescein (FAM)-TGGTCTCTAGTTCCTTCTCTAATGCTCTGA-carboxy-tetramethyl-rhodamine (TAMRA)-3'; c-kit: sense 5'-CGTGAACTCCATGTGGCTAA-3', antisense 5'-CGTCTCCTGGCGTTCATAAT-3', probe 5'-FAM-AAGCACAATAGCTGGCACCGG-TAMRA-3'; histone H2a: sense 5'-ACGAGGAGCTCAACAAGCTG-3', antisense 5'-TATGGTGGCTCTCCGTCTTC-3', probe 5'-FAM-AACATCCAGGCCGTGCTGCT-TAMRA-3'. To determine the copy number of target transcripts, cloned plasmid cDNAs for individual genes were used to generate a calibration curve. Purified plasmid cDNA templates were measured, and copy numbers were calculated based on absorbance at 260 nm. A calibration curve was created by plotting the threshold cycle against the known copy number for each plasmid template diluted in log steps from 10^6 ^to 10^1 ^copies. Each run included diluted plasmids to generate a calibration curve, a negative control without a template, and samples with unknown mRNA concentrations. Data were normalized based on histone H2a transcript levels.

### Immunohistochemistry

To localize KITL, ovaries were obtained from PMSG-primed mice at 4 h after hCG treatment. After fixation with 10% neutral formalin (pH 7.4) for 24 h, tissues were embedded in paraffin and sectioned at 6-μm intervals. After de-paraffinization and dehydration, endogenous peroxidase activities were quenched with 0.3% hydrogen peroxidase-methanol for 30 min. After blocking with 10% BSA-PBS for 30 min, slides were then incubated with goat anti-KITL antibodies characterized previously[20] (R&D system, Minneapolis, Minnesota, USA) at 1:200 dilution overnight at 4 C. After three washes in Tris-buffered saline (TBS), slides were incubated with biotinylated anti-goat secondary antibodies (Nichirei Bioscience, Tokyo, Japan) for 30 min at room temperature. After three washes, bound antibodies were visualized using a DAB kit (DAKO Corp., Carpinteria, California, USA) and tissues were counterstained with hematoxylin. For negative controls, the primary antibodies were replaced by nonimmune goat IgG (DAKO Corp.).

### Follicle cultures

Preovulatory follicles were excised from mouse ovaries at 48 h after PMSG treatment and cultured to examine nuclear maturation of oocytes [6]. Follicles (15–20 per vial) were treated with or without recombinant murine KITL (Peprotech Inc., Rocky Hill) or epidermal growth factor (EGF) (positive control, Sigma, St. Louis, MO) in Leibovitz's L-15 medium (Invitrogen). The vials were flushed at the start of the culture with O2/N2 (at a 1:1 ratio), sealed, and cultured at 37 C with gentle shaking for 8 h. After culture, COCs were isolated, and, after cumulus cell removal, oocytes are examined for the occurrence of GVBD under a Hoffman contrast microscopy (Nikon Inc., Tokyo, Japan).

### Evaluation of first polar body extrusion

Either COCs or DOs were cultured with or without KITL to evaluate the effect of KITL on the transition from metaphase I (MI) stage to metaphase II (MII) stage oocytes. The doses of KITL for the present studies were based on the levels of KITL in human follicular fluid (560–660 pg/ml)[21] and those of other ovarian paracrine factors used in oocyte maturation studies[5,7]. COCs and DOs were obtained from mouse ovaries at 48 h after PMSG-treatment following isolation of preovulatory follicles and puncturing of the largest follicles as described above. After washing, COCs or DOs were transferred to M16 medium (Sigma) without FBS, and cultured with or without KITL for 24 h at 37 C in 5% CO_2 _95% air. Some COCs were cultured with KITL with or without a neutralizing goat anti-mouse KITL antibody (R&D system). The occurrence of first polar body extrusion in the oocytes was examined after removing cumulus cells by using a small-bore pipette.

### COC cultures and immunoblotting analyses

COCs were cultured with or without KITL to measure the levels of cyclin B1 in oocytes by immunoblotting. COCs were obtained from mouse ovaries at 48 h after PMSG treatment and cultured with or without 5 ng/ml KITL as described above. After 7 h of treatment, cumulus cells were removed and the metaphase I (MI) oocytes were collected for immunoblotting. Fifty oocytes were homogenized in a buffer containing 137 mM NaCl, 20 mM Tris-HCl, 1% Nonidet P40, 10% glycerol, 100 μg/ml phenylmethylsulphonylfluoride(PMSF), and 100 μg/ml leupeptin. Samples were boiled for 3 min, and separated by electrophoresis on 12% SDS-PAGE. Prestained SDS-polyacrylamide gel electrophoresis (PAGE) standards (Invitrogen) were used as molecular weight marker. They were then transferred onto nitrocellulose membranes (Protran; Schleicher & Schuell Bioscience, Dassel, Germany) pre-incubated for 1 h in blocking buffer containing 5% nonfat dry milk. The membranes were incubated with mouse anti-cyclin B1 monoclonal antibodies (Cell Signaling Technology, Danvers, MA, 1: 2000 dilution) at 4°C overnight. As control for loading, the membranes were also incubated with a rabbit anti-β-actin polyclonal antibody (sc-1616, Santa Cruz Biotechnology, CA) at a dilution of 1:1000. Goat anti-mouse IgG and goat anti-rabbit IgG (Zhongshan, Beijing, China) was used as the secondary antibody at a dilution of 1:2000. Blots were developed using chemiluminescent substrate from the detection system (ECL; Santa Cruz Biotechnology) and exposed to Kodak film. Exposed films were scanned on a GS 800 densitometer (Bio-Rad, Hercules, CA), and the intensity of signals was measured using Quantity One software (Bio-Rad).

### In vitro maturation, fertilization, and early embryonic development

To assess the effect of KITL treatment on the cytoplasmic maturation of preovulatory oocytes, we performed in vitro maturation of oocytes followed by in vitro fertilization as described [7,22]. COCs from PMSG-primed mice were obtained in M2 medium (Sigma) supplemented with 0.1% FBS before culturing in minimum essential media (Invitrogen) supplemented with Earle's salts, 10 μg/ml streptomycin sulfate, 75 μg/ml penicillin G, and 0.1% FBS in the presence or absence of 5 ng/ml KITL at 37 C in 5% CO2 95% air. After 16 h of treatment, cumulus cells were removed and oocytes were examined and classified according to their developmental stage (GV, MI, or MII). MII oocytes were inseminated with sperm (2–3 × 10^5^/ml) from B6D2F1 males and incubated for 6 h at 37 C in 5% CO_2 _95% air. After in vitro fertilization, fertilized oocytes were recovered, washed twice, and cultured in IVF fertilization medium (COOK, Brisbane, Australia). Twenty-two hours after insemination, two-cell-stage embryos were collected and cultured in 50 μl drops of IVF cleavage medium (COOK) for 5 more days up to the blastocyst stage. Embryonic development was monitored daily under the Hoffman modulation contrast microscopy, and the development of preimplantation embryos was assessed.

### Statistical analysis

Statistical analysis was carried out by using Mann-Whitney U test for paired comparison and the one way ANOVA followed Fisher's protected least significant difference for multiple group comparison. Results were considered to be significant at P < 0.05 for all tests. Each experiment was repeated at least three times and the results are presented as the mean ± SEM.

## Results

### Gonadotropin stimulation of the ovarian expression of Kitl

**Figure 1:**
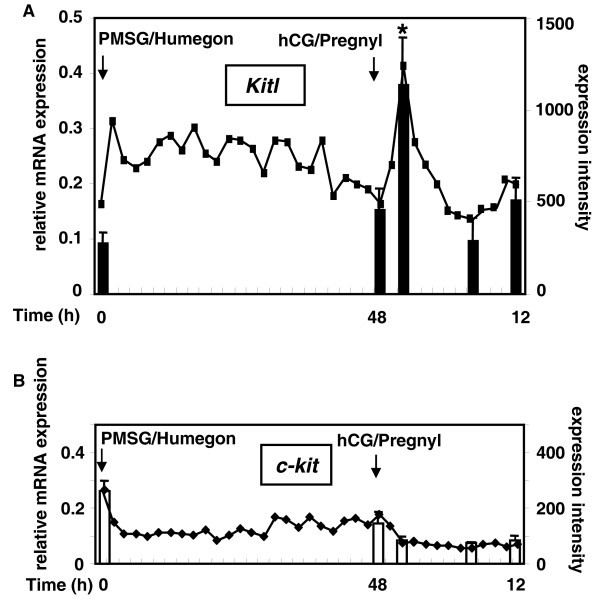
**Gonadotropin stimulation of Kitl expression in mouse ovaries**. Gonadotropin regulation of Kitl (A) and c-kit (B) transcripts in ovaries. Line graphs represent DNA microarray data depicting the expression intensity of each transcript (right y axis), whereas bar graphs depict quantitative real-time RT-PCR results (left y axis; mean ± SEM, n = 4). Values of expression intensity were derived from integration of hybridization signals from multiple probe sets for individual genes. Data were normalized based on histone H2a transcript levels. *, P < 0.05 vs. 0 h of PMSG treatment.

We used DNA microarray analyses to identify ovarian paracrine ligands induced by LH/hCG during the preovulatory period. As shown in Figure 1A (line graph), the expression of Kitl mRNA was stimulated after treatment with Pregnyl containing hCG activity, whereas the level of c-kit transcripts showed minor changes after gonadotropin treatment (Figure 1B, line graph). To confirm DNA microarray results, we performed real-time RT-PCR of ovarian transcripts for these genes in mice treated with PMSG followed by an injection of an ovulatory dose of hCG 48 h later (Figure 1, A and 1B, bar graphs). Treatment with hCG increased 4-fold Kitl transcript levels at 2 h after hormonal treatment, followed by a decline.

### Expression level of Kitl and c-kit transcripts in isolated ovarian cells and hCG stimulation of Kitl expression in granulosa cells

**Figure 2:**
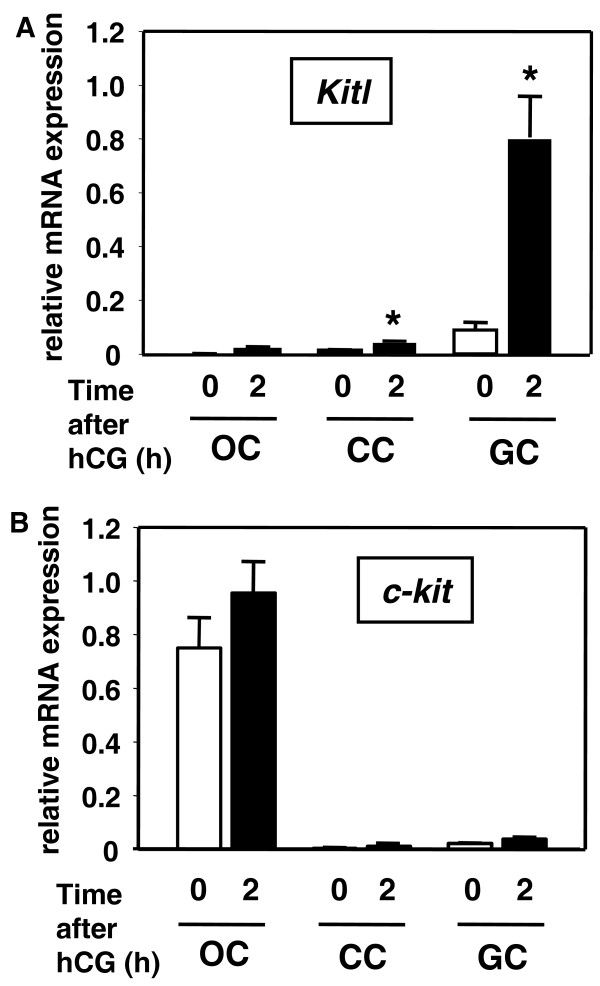
**hCG stimulation of Kitl expression in mouse cumulus and mural granulosa cells**. hCG regulation of Kitl and c-kit expression in oocytes (OC), cumulus cells (CC) and mural granulosa cells (GC) at different times before and after hCG treatment of immature mice. Transcript levels of Kitl and c-kit in isolated ovarian cells were quantified by real-time RT-PCR. (Mean ± SEM, n = 4). Data were normalized based on histone H2a transcript levels. *, P < 0.05 vs.0 h of hCG treatment.

We determined the levels of Kitl and c-kit transcripts in isolated ovarian cells by real-time RT-PCR. Kitl mRNA was expressed in both cumulus and granulosa cells, but not in oocytes (Figure 2A), whereas c-kit mRNA was predominantly expressed in oocytes among tested cell types (Figure 2B). After hCG treatment, the Kitl transcript level was increased in both cumulus and granulosa cells. Although the Kitl transcript level was significantly increased in cumulus cells, its major increase was detected in granulosa cells (Figure 2A). In contrast, the c-kit transcript level was not changed by hCG treatment (Figure 2B).

### Localization of KITL antigen in ovary

**Figure 3:**
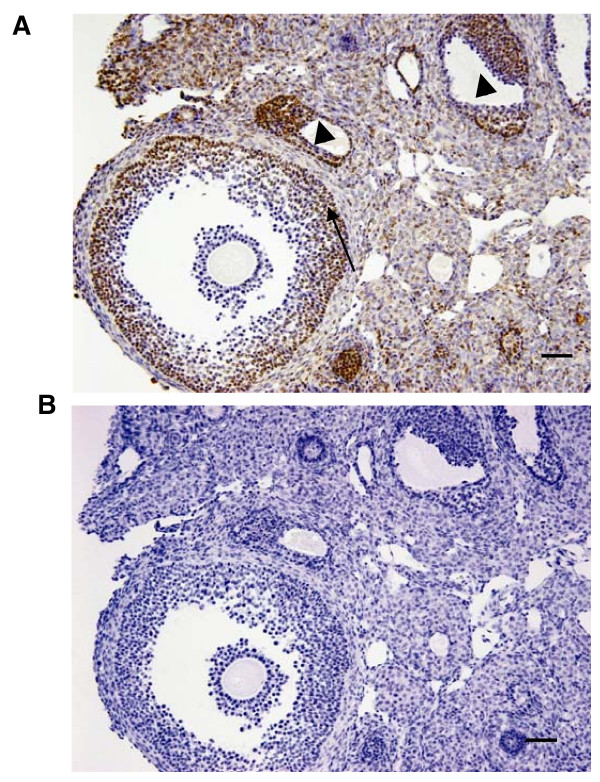
**Localization of KITL antigen in mouse ovaries**. Immunohistochemical detection of KITL in ovaries of PMSG-primed mice at 4 h after hCG injection. (A) KITL staining; (B) negative controls. KITL was detected in granulosa cells of preovulatory (arrows) and small antral follicles (arrowheads), and weaker staining of KITL was found in interstitial cells. (Scale bars, 50 μm).

We further determined the expression of KITL protein by using immunohistochemistry. As shown in Figure 3, strong KITL staining was observed in granulosa cells of both preovulatory and small antral follicles at 4 h after hCG treatment, and a weaker signal was found in interstitial cells. However, KITL protein was not detected in oocytes, cumulus cells and theca cells.

### Effects of KITL treatment on the nuclear maturation of preovulatory oocytes

**Figure 4:**
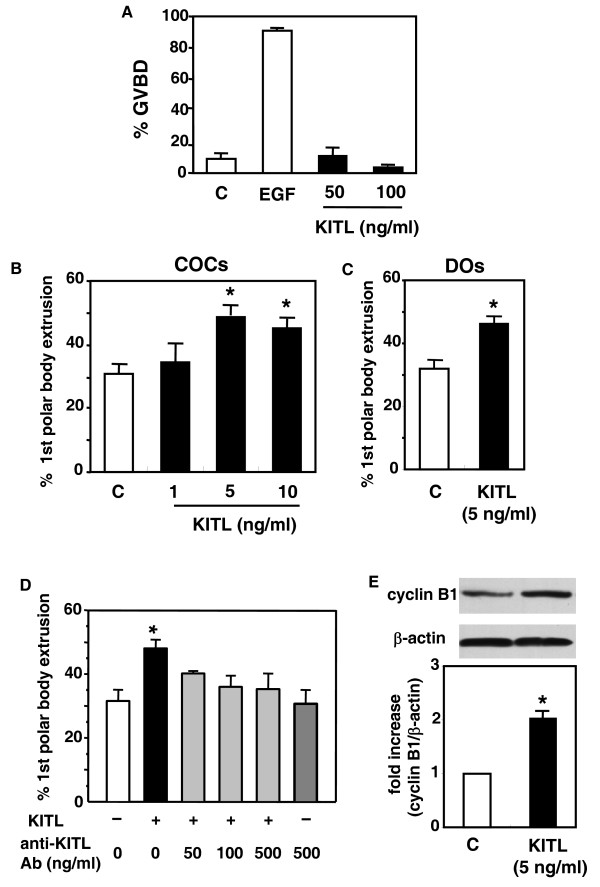
**Regulation of the nuclear maturation of preovulatory oocytes by KITL**. (A) Lack of effects of KITL on GVBD of oocytes. Preovulatory follicles were cultured without (control, C) or with EGF (2 μg/ml) or KITL (50 and 100 ng/ml) for 8 h before evaluation of oocytes undergoing GVBD. (n = 3, 42–48 follicles per experimental group). (B and C) Effects of KITL treatment on first polar body extrusion by cultured cumulus oocyte complexes (B; COCs) and denuded oocytes (C; DOs). COCs or denuded oocytes isolated from preovulatory follicles were cultured without (control, C) or with KITL for 24 h. After culture, the percentage of oocytes showing first polar body extrusion was determined (n = 6, 49–61 oocytes per experimental group). (D) Neutralizing effects of the anti-KITL antibody on KITL stimulation of first polar body extrusion. COCs were cultured with KITL (5 ng/ml) with or without the anti-KITL antibody. (n = 3, 75–79 oocytes per experimental group). E) Effects of KITL treatment on the levels of cyclin B1 protein in MI oocytes. COCs were cultured without (control, C) or with KITL (5 ng/ml) for 7 h. After removal of cumulus cells, oocytes were subjected to immunoblotting. (Upper panel) A representative immunoblot showing the cyclin B1 protein at 60 kDa and β-actin protein at 42 kDa. (Lower panel) Densitometric analyses of relative expression levels of cyclin B1 protein. The cyclin B1 levels were represented as fold increases to the control group (mean ± SEM, n = 3). *, P < 0.05 vs. control group.

Based on the hCG stimulation of Kitl expression in granulosa cells and c-kit expression in oocytes, we tested if KITL acts as a paracrine factor to regulate oocyte functions. In cultured preovulatory follicles, treatment with EGF, but not KITL, for 8 h induced GVBD in oocytes (Figure 4A). Because oocytes obtained from preovulatory follicles underwent spontaneous GVBD when cultured as COC, we used the COC model to test the effect of KITL in the promotion of further development of oocytes. As shown in Figure 4B, treatment with KITL increased first polar body extrusion of oocytes in cultured COCs in a dose-dependent manner. The proportions of first polar body extrusion in control group were 33.3%(n = 18 COCs), 40%(n = 20), 20%(n = 20), 22.2%(n = 18), 27.2%(n = 11), and 45.5%(n = 11), whereas those in 5 ng/ml KITL treated group were 46.2%(n = 13 COCs), 50%(n = 14), 36.4%(n = 15), 46.7%(n = 11), 54.6%(n = 11) and 54.6%(n = 11). Similar stimulation was evident when DOs were treated with KITL (Figure 4C), indicating a direct effect of KITL on oocyte receptors. In addition, the stimulatory effect of KITL in the COC model was blocked by cotreatment with the neutralizing anti-KITL antibody, whereas treatment with anti-KITL antibody alone was ineffective (Figure 4D).

We further assessed the molecular mechanism underlying the KITL promotion of first polar body extrusion. Because MI phase lasted between 7 and 10 h during in vitro culture in the oocytes[23], COCs obtained from preovulatory follicles were cultured for 7 h before measuring the levels of cyclin B1 proteins in oocytes at MI phase. Although cyclin B1 can be detected in all samples, its levels were increased 2.1-fold by treatment with KITL (Figure 4E).

### Effects of KITL treatment on cytoplasmic maturation

**Figure 5:**
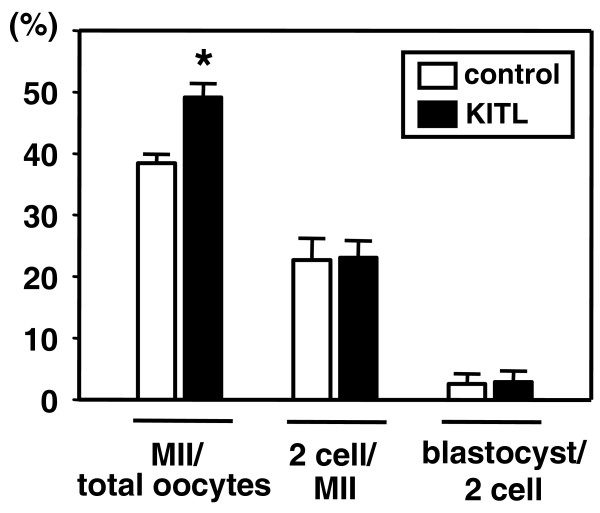
**Lack of effect of KITL in the conditioning of oocytes for development into preimplantation embryos**. COCs obtained from preovulatory follicles were cultured without (control) or with KITL (5 ng/ml) for 17 h. After progression into the MII stage, oocytes were inseminated and cultured for 5 more days without hormones. The percentage of MII oocytes developing into 2-cell-stage embryos and the percentage of 2-cell-stage embryos developing into blastocyst-stage embryos were evaluated (n = 4, 174–295 oocytes per experimental group). *, P < 0.05 vs. control group.

The role of KITL in conditioning the oocytes for subsequent fertilization and progression to blastocysts was evaluated in vitro (Figure 5). COCs obtained from mice primed for 48 h with PMSG were cultured with or without KITL. To avoid hardening of the zona pellucida that is unfavorable for in vitro fertilization, 0.1% FBS was included for all cultures. Similar to serum free cultures (Figure 4B); treatment with KITL increased the proportion of oocytes showing first polar body extrusion (Figure 5, MII oocytes). These MII oocytes were then fertilized in vitro without further treatment with KITL. However, pretreatment with KITL did not increase the proportions of MII oocytes that developed into the two-cell or blastocyst-stage embryos (Figure 5).

## Discussion

The present study demonstrates increases of Kitl expression following the preovulatory LH/hCG surge in mouse ovaries. Using isolated ovarian cells, we further showed major increase of Kitl transcripts in granulosa cells, and predominant expression of c-kit in preovulatory oocytes. In in vitro cultures, KITL directly acts on preovulatory oocytes to stimulate first polar body extrusion, but not GVBD and cytoplasmic maturation.

Previous studies on the control of oocyte maturation have focused mainly on endocrine regulation of the hypothalamic-pituitary-ovarian axis. However, it has been recognized that intraovarian factors are also important in regulating oocyte maturation [24]. The preovulatory LH surge triggers a cascade of events in ovarian follicles, including the resumption of meiotic maturation, luteinization, expansion or maturation of the cumulus cells, and follicle rupture. However, oocytes express few or no LH receptors and are insensitive to direct LH stimulation [25]. Thus, the LH-responsive granulosa or theca cells could secret paracrine factors to regulate oocyte functions. To identify such intraovarian factors important for oocyte maturation, we performed DNA microarray analyses of the ovarian transcriptome during the peri-ovulatory period to provide a genome-wide screen of candidate genes. Here, we identified increases in the expression of Kitl mRNA after hCG stimulation in whole ovaries and isolated ovarian granulosa cells. Although Kitl mRNA was also increased in cumulus cells following hCG treatment, its level was lower than that of granulosa cells. Furthermore, KITL protein could be detected in granulosa, but not cumulus cells of preovulatory follicles obtained from ovaries of PMSG-primed mice at 4 h after hCG treatment. Granulosa cells in preantral and early antral follicles also expressed KITL, suggesting diverse role of KITL and c-kit throughout folliculogenesis and oogenesis, including the establishment of primordial germ cells within the ovary, primordial follicle activation, oocyte survival and growth, granulosa cell proliferation, theca cell recruitment, et al. The observed increases of Kitl mRNA in whole ovaries and granulosa cells are consistent with an earlier report using PMSG-primed rats at 6 h after hCG treatment [26]. Likewise, low expression of Kitl was shown in cumulus cells of mouse antral follicles [8]. Our findings of the predominant expression of c-kit mRNA in preovulatory oocytes are also consistent with earlier reports [8,27,28]. Because c-kit is also expressed in theca cells of antral follicles, corpora lutea, and some interstitial cells of the ovary [29], paracrine actions of KITL on the these cells cannot be ruled out.

Completion of the nuclear maturation of oocytes involves GVBD and extrusion of the first polar body. Although treatment of cultured COCs with KITL did not affect GVBD of preovulatory oocytes, it facilitated first polar body extrusion by 55% vs. control groups without treatment. This effect is comparable to that of another ovarian factor, IGF-I, showing 57% increases in first polar body extrusion vs. controls [30]. The dose range of KITL used in the present studies was similar to that used in the rat study[26,31]. Furthermore, the concentrations of KITL in human preovulatory follicular fluid were comparable to those levels[21]. Similar to the BDNF stimulation of first polar body extrusion, but not GVBD, it is apparent that sequential steps of nuclear maturation of the oocyte are controlled by different paracrine factors. Although we detected transcripts of c-kit in both cumulus cells and oocytes in COCs, KITL induced first polar body extrusion in denuded oocytes, suggesting its direct effects on oocytes. Cyclin B is the regulatory subunit of maturation promoting factor, which induces meiotic resumption of immature mammalian oocytes[32]. Mouse oocytes do not require de novo synthesis of proteins to undergo GVBD in vitro, whereas the synthesis of cyclin B1 is indispensable for the progression of meiotic maturation after GVBD. Rate of cyclin B1 synthesis controls the length of the first meiotic M phase[33]. Our data on the increase in cyclin B1 protein in MI oocytes following KITL treatment suggest that cyclin B1 is involved in KITL promotion of completion of meiosis I.

Although a previous study reported that treatment of isolated preovulatory oocytes with KITL transiently blocked GVBD [26,31], we did not observe an effect of KITL on GVBD of preovulatory oocytes using a follicle culture model. Because isolated oocytes undergo spontaneous GVBD after removal from surrounding somatic cells without LH treatment, our studies utilizing the preovulatory follicle model without spontaneous GVBD of the oocyte are more likely to reflect the in vivo conditions. Recent study demonstrated oocyte growth was limited in the absence of KITL, and yet spontaneous GVBD which could be inhibited by KITL2 was observed in these oocytes. The spontaneous GVBD is regarded as premature resumption of meiosis that reflects oocyte degeneration, rather than the physiologic meiosis [10]. Their findings further supported our results. Our unpublished data further indicated that KITL treatment did not induce cumulus cell expansion in preovulatory follicles cultured with the serum-free medium. Mutant mice with defects in KITL or c-kit showed similar phenotypes including impairment of primordial germ cell survival, migration and proliferation, as well as defective follicle development [8]. Because these mutant mice exhibit developmental arrest of follicles at primary stage, no female mice were available for investigating the roles of KITL/c-kit signaling in the preovulatory follicles. Thus, the roles of endogenous KITL in the oocyte maturation remain to be determined.

Some oocytes competent to complete nuclear maturation are unable to develop into blastocyst stage, which is indicative of deficient or defective cytoplasmic maturation of the oocyte [3]. During the maturation process, oocytes undergo profound cytoplasmic changes which are essential for normal fertilization and embryonic development. However, treatment of preovulatory oocytes with KITL did not improve the developmental capacity of mouse oocytes. Our data is consistent with previous findings demonstrating that the concentrations of KITL in the human follicular fluid did not associate with the fertilization rate and pregnant outcome in patients undergoing intracytoplasmic sperm injection [21]. Thus, the processes of nuclear and cytoplasmic maturation of the oocyte are independently regulated and could be controlled by different paracrine factors.

It has been demonstrated that there are significant interactions between oocyte secreted factors and KITL[17]. Growth differentiation factor (GDF9) from oocyte has been shown to inhibit kitl expression in granulosa cells[34] while present study demonstrates the enhancement of nuclear maturation by KITL produced by granulosa cells. And recent studies indicated an important role of GDF9 in promoting oocyte developmental potential [35-37]. These studies suggest that there are complex regulatory loops between oocytes and follicular somatic cells. Further study is needed to clarify the role of KITL and GDF9 interaction in oocyte nuclear and cytoplasmic maturation.

## Conclusion

In the present study, we demonstrated increases of Kitl expression following LH/hCG stimulation in preovulatory granulosa cells and the ability of KITL to promote the first polar body extrusion of preovulatory oocytes. It is becoming clear that this ligand signaling system is important for diverse ovarian processes including primodial germ cell endowment, primordial follicle activation, theca cell recruitment, the antrum formation, and oocyte growth [8]. The present demonstration of an augmenting role of KITL for the nuclear maturation of preovulatory oocytes underscores the importance of diverse paracrine systems for oocyte maturation. Understanding of these intercellular communication networks could lead to new approaches in the treatment of infertility, and facilitate future formulation of the optimal culture conditions for in vitro maturation and in vitro fertilization of oocytes.

## Competing interests

The authors declare that they have no competing interests.

## Authors' contributions

YY performed all experiments with following coauthors helps. KK participated in the study design and supported the experiments of real-time PCR, western blotting and cell culture. MS and NK helped to performed the experiments of real-time PCR. PG and MDSG carried out the analysis of DNA microarray. RR supported the cell culture. AJWH conceived of the study, and participated in its design and helped to draft the manuscript. TT participated in study design and helped to draft the manuscript. All authors read and approved the final manuscript.
